# Photoacclimation strategies in northeastern Atlantic seagrasses: Integrating responses across plant organizational levels

**DOI:** 10.1038/s41598-018-33259-4

**Published:** 2018-10-04

**Authors:** Nadine Schubert, Cátia Freitas, André Silva, Monya M. Costa, Isabel Barrote, Paulo A. Horta, Ana Claudia Rodrigues, Rui Santos, João Silva

**Affiliations:** 1 Programa de Pós-graduação em Oceanografia, Centro de Ciências Físicas e Matemáticas, Universidade Federal de Santa Catarina, Campus Trindade, Florianópolis, Brazil; 2 CCMAR - Centre of Marine Sciences, University of Algarve, Campus Gambelas, 8005-139 Faro, Portugal; 3 Departamento de Botânica, Centro de Ciências Biológicas, Universidade Federal de Santa Catarina, Campus Trindade, Florianópolis, Brazil

## Abstract

Seagrasses live in highly variable light environments and adjust to these variations by expressing acclimatory responses at different plant organizational levels (meadow, shoot, leaf and chloroplast level). Yet, comparative studies, to identify species’ strategies, and integration of the relative importance of photoacclimatory adjustments at different levels are still missing. The variation in photoacclimatory responses at the chloroplast and leaf level were studied along individual leaves of *Cymodocea nodosa, Zostera marina* and *Z. noltei*, including measurements of variable chlorophyll fluorescence, photosynthesis, photoprotective capacities, non-photochemical quenching and D1-protein repair, and assessments of variation in leaf anatomy and chloroplast distribution. Our results show that the slower-growing *C. nodosa* expressed rather limited physiological and biochemical adjustments in response to light availability, while both species of faster-growing *Zostera* showed high variability along the leaves. In contrast, the inverse pattern was found for leaf anatomical adjustments in response to light availability, which were more pronounced in *C. nodosa*. This integrative plant organizational level approach shows that seagrasses differ in their photoacclimatory strategies and that these are linked to the species’ life history strategies, information that will be critical for predicting the responses of seagrasses to disturbances and to accordingly develop adequate management strategies.

## Introduction

Seagrasses live in highly dynamic environments, characterized by significant fluctuations in the amount of available light. As such, they have evolved a number of acclimatory responses that occur at different organizational levels and over periods of time ranging from seconds to seasons. There is a substantive body of literature reporting different seagrass photoacclimatory responses that indicate species-specific capacities and strategies. Yet, the vast majority of studies have focused on a single species or single plant organizational level. Hence, comparative studies on acclimatory responses of different seagrass species, as well as studies integrating the relative importance of adjustments at different organizational levels, are needed. The latter represents a concept widely applied in terrestrial plant science to define the ‘acclimation potential’ and strategy of species^1,2^. Applying it to seagrasses will allow recognizing how diverse they are with respect to their photoacclimatory responses/strategies and in their tolerance to rapidly changing light environments, as depending on the strategy adopted, the timing of the species’ response will differ.

Numerous studies in seagrasses have addressed the adjustments at single organizational scales, including chloroplast, leaf and shoot/meadow level responses^3–7^. Chloroplast-level (or physiological and biochemical) responses to variation in the light environment are related to changes in the photosynthetic and photoprotective capacity and pigment concentrations^8,9^. Leaf-level responses include changes in morphology (length, width, thickness), in area per unit of leaf biomass (SLA- specific leaf area) and number of chloroplasts per unit leaf area, which are associated with changes in chlorophyll content and photosynthetic capacity per unit leaf area^10–13^. At the shoot/meadow-level, seagrasses adjust to varying light regimes through changes in leaf biomass and shoot height and density, which usually increase with light intensity, thereby reducing the light reaching individual plants^10,13–15^. Such light gradients within seagrass meadows result in light reaching individual seagrass leaves and leaf sections that may vary several orders of magnitude^15–17^, causing significant differences in the photosynthetic performance of plants from meadows with different shoot densities and/or leaf biomass, as well as along individual leaves.

Many observations of photoacclimatory responses of northeastern Atlantic seagrasses have been reported, ranging from changes in leaf biomass and/or shoot density, variations in leaf morphology, to physiological and biochemical adjustments (Table 1). Under high light intensities, both *Cymodocea nodosa* and *Zostera marina* generally increased their leaf biomass, while decreasing shoot height, resulting in shorter, narrower and thinner leaves, even though a few studies reported the opposite response in *Z. marina*. On the other hand, the chloroplast-level responses of photosynthetic parameters and pigment concentration of *C. nodosa* are highly variable, whereas in *Z. marina* an increase in photosynthesis and a decrease in chlorophyll content has been described (Table 1). In contrast with *Z. marina*, only a few studies are available on the subject for its congeneric *Z. noltei*, a mostly intertidal species^18^. Similar to its sister species, this species responds with increasing shoot density in response to higher light conditions, while there are contrasting reports on the changes in leaf morphology and photosynthetic capacity (Table 1).

**Table 1:** Summary of reported photoacclimatory responses (high-light adjustments compared to low light) at different plant organizational levels of northeastern Atlantic seagrass species.

Species		Meadow/shoot level	Leaf level	Chloroplast level	Refs
*Cymodocea nodosa*	D		↓ Leaf length, width and thickness ↑ SLA	=P_max_, α ↑ Respiration, ↓ [Chl]	^12^
	D	↑ Leaf biomass, shoot density		↓ P_max_, α, [Chl]	^23^
	D	↑ Leaf biomass		=ETR_max_ ↓ α_ETR_, [Car]	^51^
	E			↑ P_max_, ↓ α =DPS	^8^
	E	↓ Shoot height and growth = Leaves per shoot	↑ SLA	↑ P_max_, α, P/R, [Chl], [Car] =Respiration, Chl*a*/Chl*b*	^52^
*Zostera marina*	D	↓ Shoot height		↓ [Chl]	^53^
	D	↑ Shoot density	↓ Leaf length and width		^54^
	D	↑ Shoot density	↓ Leaf length and width	↑ ETR_max_, NPQ, [Car] ↓ α	^13^
	D	↓ Shoot length ↑ Leaf biomass		↑ P_max_ ↓ Respiration, [Chl]	^19^
	D	↓ Leaf biomass		↑ P_max_, Respiration, ↓ [Chl]	^34^
	E	↑ LAI	↑ Leaf length		^55^
	E			↑ P_max,_ α, DPS	^8^
	E		↑ Leaf length and width	↑ ETR_max_	^56^
	E			↑ P_max_, α, Respiration, ↓ [Chl]	^57^
*Zostera noltei*	D			↑ ETR_max_	^4^
	E			= ETR_max_	^58^
	E	↑ Shoot density			^59^
	E		↑ Leaf length and width		^60^
	E		↓ Leaf length		^61^

(D- different depths, E- experimental changes in light levels; LAI- leaf area index; SLA- specific leaf area; P_max_, ETR_max_- maximum photosynthetic and electron-transport rate; α- photosynthetic efficiency; [Chl], [Car]- chlorophyll and carotenoid concentration; DPS- de-epoxidation state).

These photoacclimatory responses of seagrasses derive mostly from single-species or single–organizational level studies, making it difficult to integrate responses within a species or to compare photoacclimatory strategies among species. The goal of this study was to determine and compare the photoacclimatory responses of the three North Atlantic seagrass species, *C. nodosa, Z. marina* and *Z. noltei*, specifically focusing on the adjustments at the chloroplast and leaf level. We examined physiological, biochemical and anatomical adjustments in response to canopy light gradients along individual leaves. Integrating the adjustments at the different levels, the capacity and strategies of the species to cope with light changes were evaluated and discussed in relation to the species’ life history strategies.

## Results

### Shoot density and Leaf Area Index (LAI)

*Cymodocea nodosa* and *Z. marina* meadows showed similar shoot densities (288 ± 41 and 236 ± 29 shoots m^−2^, respectively), while *Z. noltei* meadow was significantly denser (p < 0.0001), with twice as many shoots per square meter (588 ± 91 shoots m^−2^). The species also differed in the number of leaves per shoot (three leaves in *C. nodosa* and *Z. noltei*, four leaves in *Z. marina*) and leaf size, with longer and wider leaves of *C. nodosa* and *Z. marina* (30–33 cm long, 0.5–0.6 cm wide), compared to *Z. noltei* (24 cm long, 0.2 cm wide). This resulted in differences in LAI, which was lowest in *Z. noltei* (LAI = 0.50 m^2^ m^−2^), followed by *C. nodosa* (LAI = 0.7 m^2^ m^−2^) with the highest value found for *Z. marina* (LAI = 1.04 m^2^ m^−2^).

### Seagrass light absorption and photosynthesis

**Figure 1:**
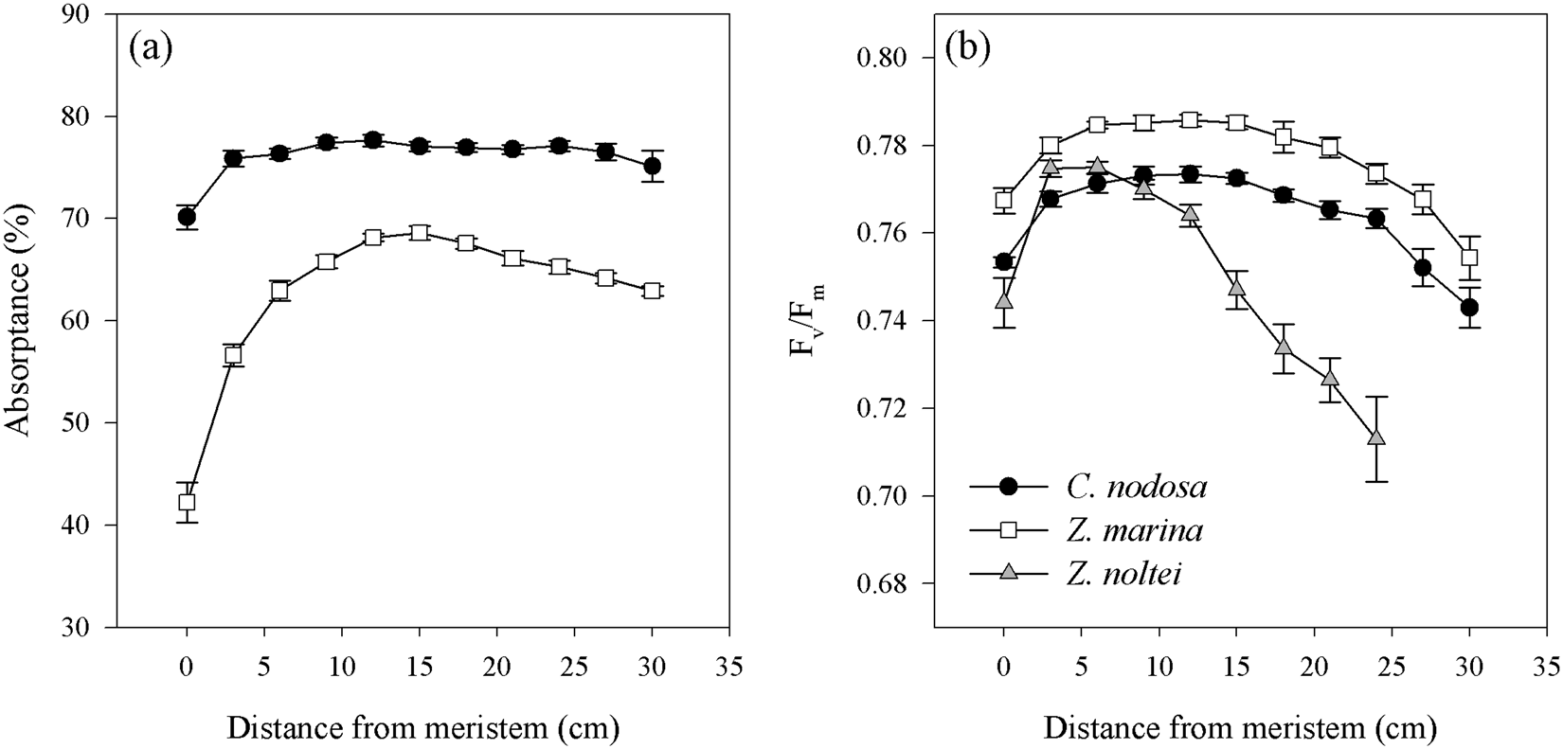
Variation of absorptance (**a**) and F_v_/F_m_ (**b**) along the 2^nd^ youngest leaf of *C. nodosa*, *Z. marina* and *Z. noltei*. Absorptance along *Z. noltei* leaves could not be measured (see Material and Methods). Data represent mean ± SE, n = 20).

*Cymodocea nodosa* exhibited higher light absorptance values (up to 77%) than *Z. marina* (Fig. 1a). In both species, absorption was lower near the meristem than in the rest of the leaf. Whereas the spatial variation in absorptance along *C. nodosa* leaves was minimal, in *Z. marina* there was a strong increase in light absorption from the basal to the middle section (from 42 to 69%), with a subsequent slight decline towards the leaf tip (Fig. 1a).

*Zostera marina* showed higher values of F_v_/F_m_ along the leaves than *C. nodosa*, but with a similar variation pattern. F_v_/F_m_ increased from the base of the leaf to its middle, with the highest values between 6 and 15 cm, and decreased towards the tip, (Fig. 1b). In *Z. noltei*, the initial increase of F_v_/F_m_ was higher, the highest values were observed in a shorter section (3–6 cm) and the subsequent drop towards the leaf tip was more pronounced (Fig. 1b).

**Figure 2:**
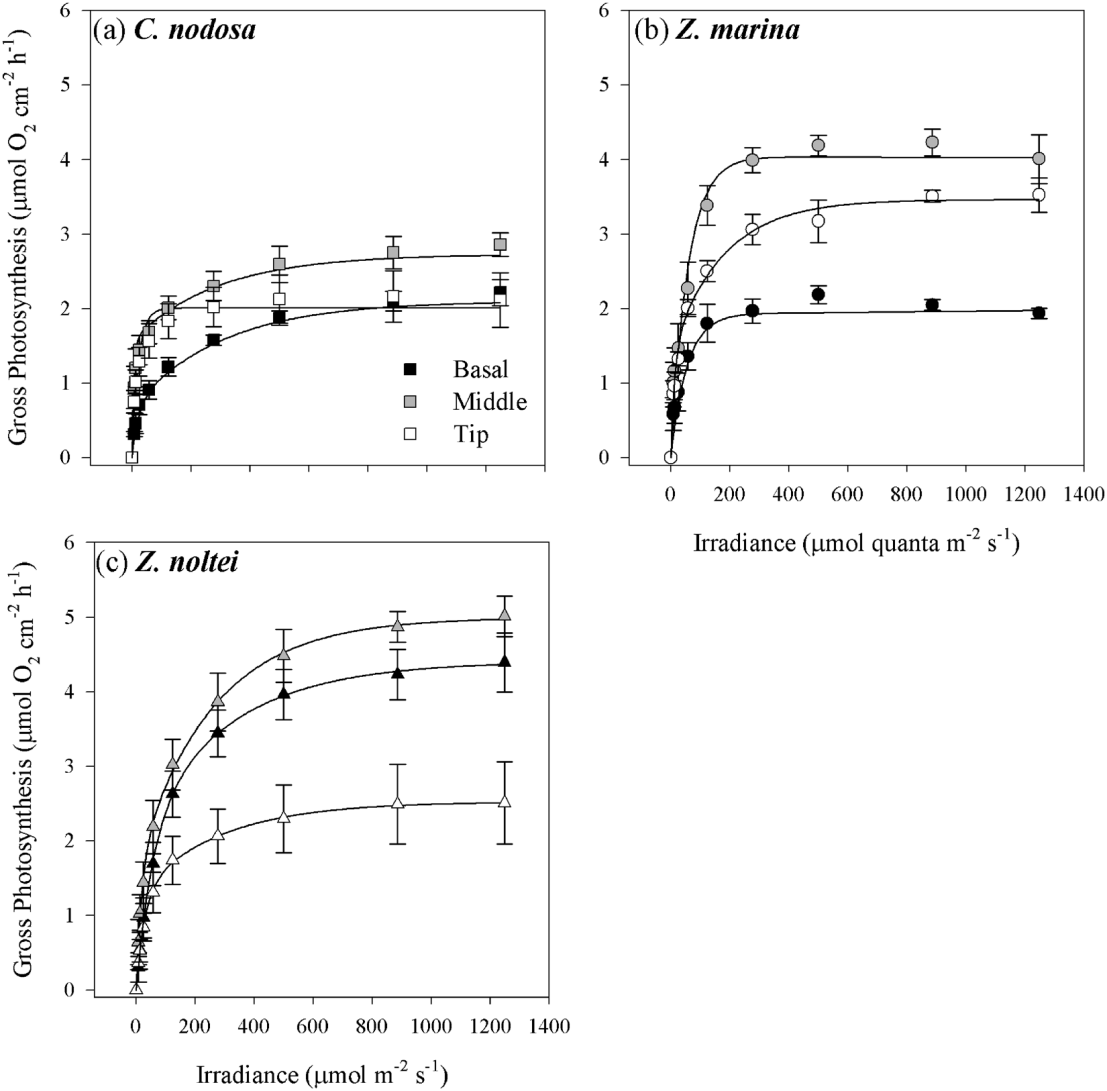
Photosynthesis-Irradiance curves performed on different leaf sections from 2^nd^ youngest leaves of *C. nodosa* (**a**), *Z. marina* (**b**) and *Z. noltei* (**c**). Data represent mean ± SE (n = 5).

Different patterns of the photosynthetic characteristics along the leaves were found in the three studied species. As expected, the middle leaf section, which also had the highest F_v_/F_m_ values (Fig. 1), exhibited the highest maximum photosynthetic rates (P_max_) in all species, when normalized by surface area, while different patterns were found with respect to the basal section and the leaf tip (Fig. 2; Table 2). In *C. nodosa*, the leaf tip did not exhibit significant differences to the basal section of the leaf, while in *Z. marina* the leaf tip showed intermediate P_max_ values, with the lowest values in the basal section (Table 2). A different pattern was shown in *Z. noltei*, where P_max_ was lowest at the leaf tip, without significant differences between middle and basal section (Table 2). The photosynthetic efficiency (α) did not vary along the leaves of the three species and in the case of the irradiance of compensation (I_c_) differences between leaf sections were found only in *Z. marina*, with significantly lower values in the leaf tip, while there were no differences along the leaves of the species for dark respiration and saturation irradiance (I_k_) (Table 2).

**Table 2:** Photosynthetic parameters (n = 5), normalized by surface area and dry weight, and chlorophyll content (μg cm^−2^, n = 6) of *C. nodosa*, *Z. marina* and *Z. noltei* leaf sections.

	P_max_		α		R_D_		I_c_	I_k_	Chl*a*	Total Chl	Chl*a*/Chl*b*
	per area	per DW	per area	per DW	per area	per DW					
*C. nodosa*											
Tip	2.1 ± 0.3^a^	414 ± 49^a^	0.042 ± 0.006^a^	8.3 ± 0.9^a^	0.79 ± 0.15^a^	171 ± 18^a^	19 ± 5^a^	53 ± 6^a^	24.2 ± 4.1^a^	33.4 ± 5.5^a^	2.7 ± 0.1^a^
Middle	2.8 ± 0.2^b^	384 ± 29^a^	0.045 ± 0.002^a^	6.7 ± 0.3^a^	0.86 ± 0.07^a^	117 ± 8.0^b^	16 ± 2^a^	64 ± 7^a^	26.4 ± 2.8^a^	35.7 ± 3.8^a^	2.9 ± 0.1^a^
Basal	2.2 ± 0.2^a^	258 ± 30^b^	0.035 ± 0.003^a^	4.2 ± 0.6^b^	1.00 ± 0.14^a^	109 ± 13^b^	28 ± 6^a^	64 ± 6^a^	24.1 ± 5.6^a^	32.6 ± 3.5^a^	2.9 ± 0.1^a^
*Z. marina*											
Tip	3.2 ± 0.3^a^	967 ± 83^a^	0.025 ± 0.004^a^	6.8 ± 0.8^a^	0.36 ± 0.08^a^	99 ± 23^a^	11 ± 1^a^	96 ± 9^a^	20.2 ± 1.2^a^	27.3 ± 1.7^a^	2.9 ± 0.1^a^
Middle	4.1 ± 0.3^b^	1134 ± 43^b^	0.031 ± 0.006^a^	6.9 ± 0.9^a^	0.51 ± 0.34^a^	170 ± 73^a^	26 ± 6^b^	113 ± 26^a^	30.1 ± 1.9^b^	41.2 ± 2.9^b^	2.8 ± 0.1^a^
Basal	2.1 ± 0.1^c^	546 ± 41^c^	0.017 ± 0.002^a^	4.2 ± 0.4^b^	0.43 ± 0.06^a^	94 ± 20^a^	25 ± 3^b^	108 ± 9^a^	15.7 ± 0.8^c^	21.4 ± 1.1^c^	2.8 ± 0.1^a^
*Z. noltei*											
Tip	2.5 ± 0.5^a^	693 ± 118^a^	0.028 ± 0.004^a^	7.9 ± 0.9^a^	1.3 ± 0.4^a^	395 ± 122^a^	37 ± 13^a^	100 ± 13^a^	19.2 ± 2.1^a^	24.7 ± 2.8^a^	3.6 ± 0.1^a^
Middle	4.9 ± 0.2^b^	963 ± 52^b^	0.048 ± 0.006^a^	9.2 ± 0.7^a^	1.4 ± 0.4^a^	272 ± 63^a^	29 ± 5^a^	106 ± 12^a^	31.7 ± 2.4^b^	40.7 ± 3.2^b^	3.6 ± 0.1^a^
Basal	4.3 ± 0.4^b^	883 ± 11^ab^	0.045 ± 0.008^a^	9.2 ± 1.3^a^	1.7 ± 0.4^a^	334 ± 51^a^	37 ± 7^a^	100 ± 12^a^	22.6 ± 1.8^a^	29.4 ± 2.2^a^	3.4 ± 0.1^a^

Data represent mean ± SE and different superscript letters indicate statistical differences between leaf sections within the same species (one-way ANOVA, *P* < 0.05, Newman-Keuls). (Maximum gross photosynthesis (P_max_) and dark respiration (R_D_) in μmol O_2_ cm^−2^ h^−1^ or μmol O_2_ g DW^−1^ h^−1^, α-photosynthetic efficiency in μmol O_2_ cm^−2^ h^−1^ [μmol quanta m^−2^ s^−1^]^−1^ or μmol O_2_ g DW^−1^ h^−1^ [μmol quanta m^−2^ s^−1^]^−1^, I_c_ and I_k_ - compensatory and saturating light intensity, respectively, in μmol quanta m^−2^ s^−1^).

The pattern of variation in photosynthetic characteristics along the leaves changed, when the data were normalized by dry weight, especially in the case of *C. nodosa* (Table 2). Here, P_max_ and α increased from the base to the leaf tip, with significant higher values in the leaf middle and tip, with the leaf tip also showing the highest respiration rates (Table 2). In the case of *Z. marina*, normalization by dry weight did not change the along-the-leaf patterns, while *Z. noltei* showed slight differences in the variation of P_max_. In the last, P_max_ (when normalized by dry weight) was highest in the leaf middle, but not significantly different from the values determined in the leaf base (Table 2).

Leaf chlorophyll content exhibited higher concentrations in the middle leaf section in both *Z. marina* and *Z. noltei*, when compared to the leaf tip and basal section, while it did not show variation along *C. nodosa* leaves (Table 2). The Chl*a*/Chl*b* ratios did not vary along the leaf in any of the species (Table 2).

### Photoprotective capacity and xanthophyll cycling

**Figure 3:**
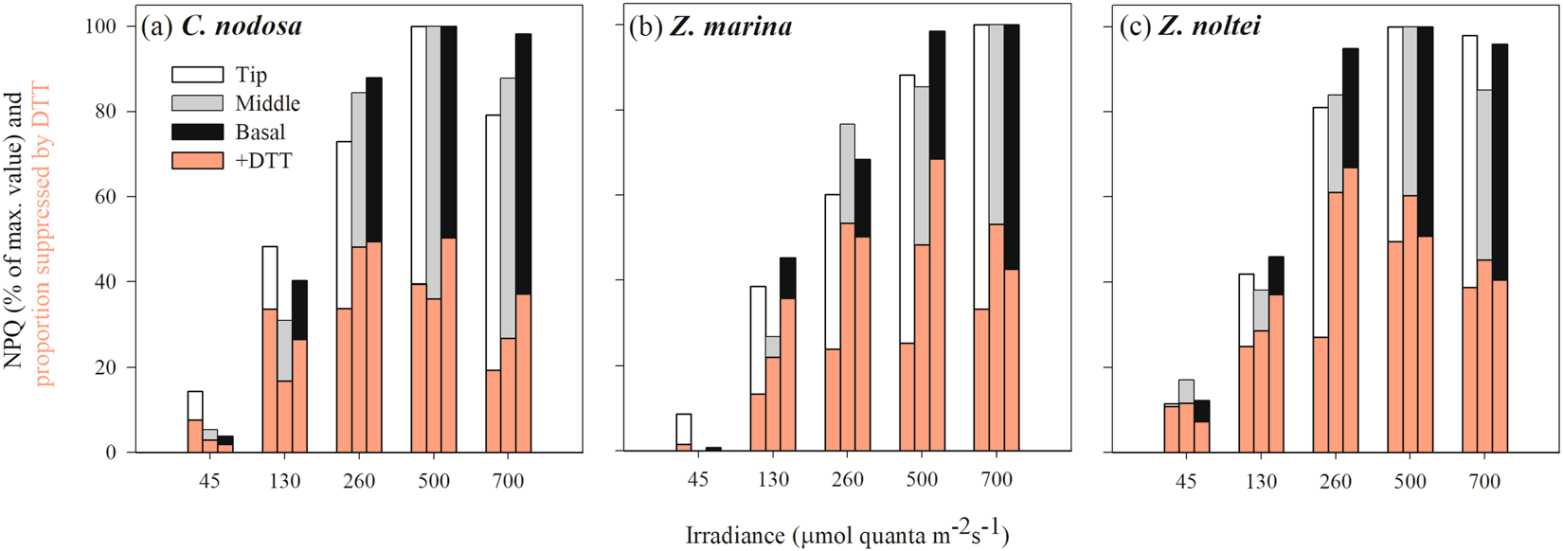
Induction of NPQ in response to increasing light intensities (expressed in % of maximum NPQ induction) in different leaf sections of *C. nodosa* (**a**), *Z. marina* (**b**) and *Z. noltei* (**c**), and the importance of xanthophyll cycling in NPQ induction (shown as % of NPQ suppressed in presence of DTT, an inhibitor of the de-epoxidation of violaxanthin to antheraxanthin, see overlying pink bars).

The mean leaf photoprotective capacity, measured here as Non-Photochemical Quenching (NPQ), differed significantly among species (P < 0.0001), with *C. nodosa* showing the lowest NPQ_max_ values and the slowest NPQ induction rate among species, without any differences along the leaf (Table 3). This agrees with its significantly smaller mean leaf VAZ-pool size and lower DPS values (P < 0.0001), compared to both *Zostera* species (Table 3). On the other hand, in the two *Zostera* species, NPQ_max_ and its induction rates were similar but in both the leaf tip achieved significantly lower NPQ_max_ values compared to the basal and middle leaf section (Table 3). Within species, no clear pattern was found between NPQ_max_ in different leaf sections and their respective photoprotective xanthophyll pigment concentrations (VAZ)-pool size and/or de-epoxidation state (DPS) values. This inconsistency between NPQ_max_ and xanthophyll cycle (XC)-pool size and DPS might be related to differences in the dependence of NPQ on the XC among species and leaf sections, here determined by using dithiothreitol (DTT) to inhibit the conversion of violaxanthin into zeaxanthin (Fig. 3).

**Table 3:** Photoprotective capacities of northeastern Atlantic seagrass species.

	NPQ_max_	NPQ_ind_ (min^−1^)	VAZ/Chl*a* (mmol mol^−1^)	Zea/Chl*a* (mmol mol^−1^)	DPS
*C. nodosa*					
Tip	3.5 ± 0.2^a^	0.15 ± 0.02^a^	118.6 ± 5.2^a^	57.1 ± 2.7^a^	0.57 ± 0.01^a^
Middle	3.1 ± 0.1^a^	0.14 ± 0.01^a^	111.6 ± 5.8^a^	46.8 ± 2.5^a^	0.52 ± 0.01^a^
Basal	3.4 ± 0.1^a^	0.13 ± 0.02^a^	124.9 ± 4.9^a^	55.8 ± 4.5^a^	0.55 ± 0.03^a^
*Z. marina*					
Tip	3.6 ± 0.2^a^	0.38 ± 0.03^a^	155.2 ± 7.7^a^	97.8 ± 4.3^a^	0.69 ± 0.01^a^
Middle	4.3 ± 0.2^b^	0.31 ± 0.01^a^	138.4 ± 5.9^a^	87.2 ± 6.0^a^	0.69 ± 0.02^a^
Basal	4.5 ± 0.2^b^	0.34 ± 0.05^a^	180.4 ± 5.5^b^	127.2 ± 6.0^b^	0.76 ± 0.02^b^
*Z. noltei*					
Tip	3.7 ± 0.1^a^	0.45 ± 0.03^a^	164.1 ± 2.7^a^	108.9 ± 6.2^a^	0.71 ± 0.02^a^
Middle	4.2 ± 0.1^b^	0.29 ± 0.04^a^	148.5 ± 8.7^b^	92.8 ± 10.3^a^	0.72 ± 0.04^a^
Basal	3.9 ± 0.2^ab^	0.35 ± 0.06^a^	136.4 ± 5.3^b^	94.2 ± 4.4^a^	0.74 ± 0.01^a^

Data represent mean ± SE (n = 6) and different superscript letters indicate statistical differences between leaf sections within the same species (one-way ANOVA, *P* < 0.05, Newman-Keuls). (Maximum Non-Photochemical Quenching (NPQ_max_), NPQ fast induction rates (NPQ_ind_), photoprotective xanthophyll pigment concentrations (VAZ), zeaxanthin (Zea) content, and de-epoxidation state of the xanthophyll cycle (DPS)).

NPQ in *C. nodosa* was suppressed by ~50–70% in the presence of DTT in all leaf sections upon exposure of up to 260 μmol m^−2^ s^−1^, while the dependence of NPQ on XC decreased at higher light intensities (25–50%; Fig. 3a). In *Z. marina*, NPQ in the leaf tip depended much less on XC, compared to the other leaf sections, as here only 18–40% of NPQ were suppressed by DTT, but was induced already at an irradiance < I_k_ (Fig. 3b). A similar pattern was also found in *Z. noltei* (NPQ suppression in leaf tip = 34–50%, in the middle and basal sections = 42–81%). However, in contrast to *Z. marina*, NPQ in *Z. noltei* was induced in all leaf sections at sub-saturating light intensity (<I_k_), where it depended greatly on XC (60–94%, depending on leaf section) (Fig. 3c).

### Photodamage repair

**Figure 4:**
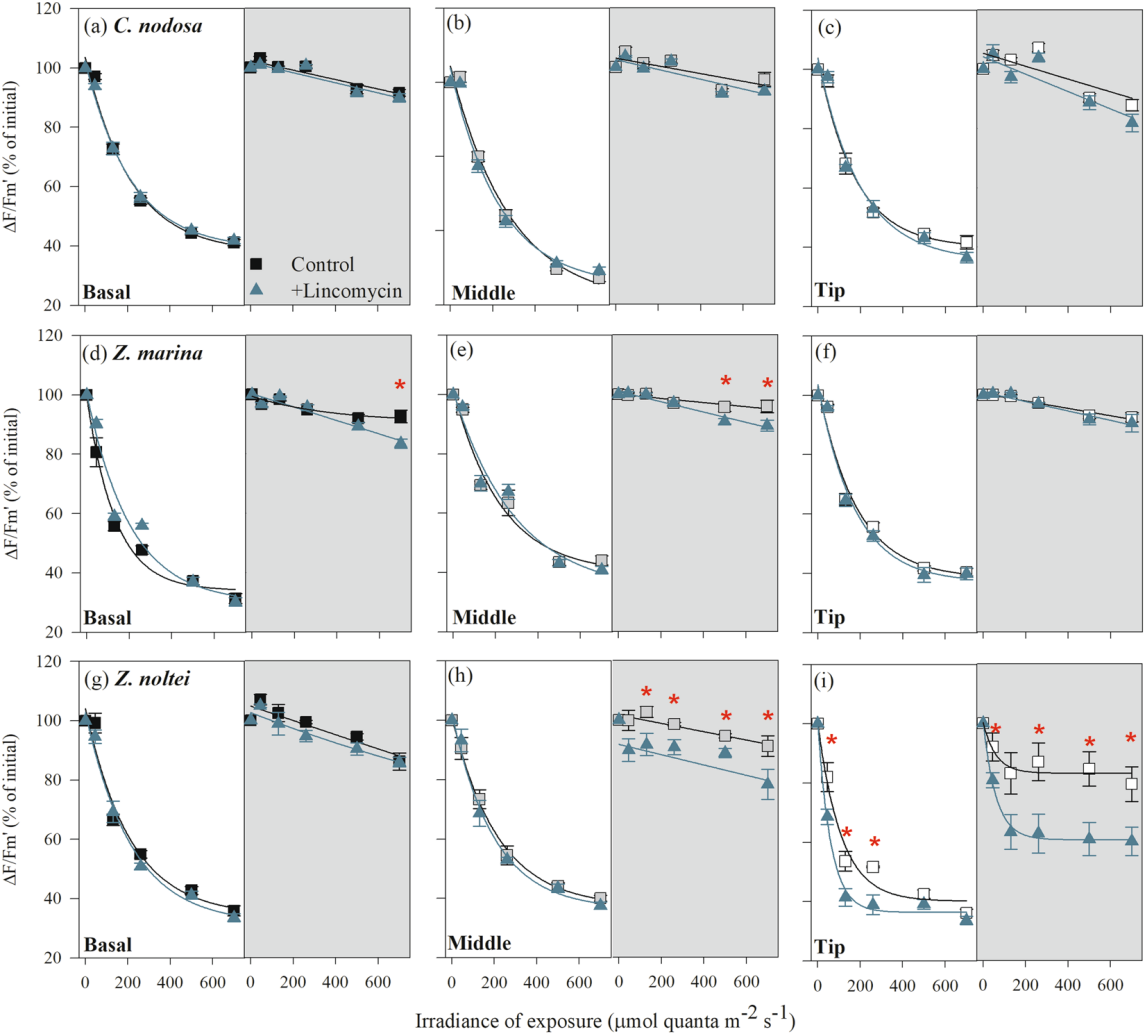
Response of ΔF/F_m′_ to high light exposure (indicated by white background) and after 1 h of recovery under dim-light conditions (indicated by grey background) in the absence (squares) and presence of lincomycin (inhibitor of D1 protein repair; blue triangles) in different leaf sections (white squares-leaf tip; grey squares-middle section; black squares-basal section) of *C. nodosa* (**a**–**c**), *Z. marina* (**d**–**f**) and *Z. noltei* (**g**–**i**). Data represent mean ± SE (n = 6) and significant differences between control and lincomycin-treatment (*P* ≤ 0.05) are indicated by asterisks.

When exposed to a series of increasing light intensities, the responses of the three species, as well as their leaf sections, showed relatively similar patterns. In all species, ΔF/F_m′_ decreased exponentially with increasing light intensity after 1 h of light exposure, while it showed a negative linear relationship with the light intensity of exposure after 1 h of recovery. The exception was the leaf tip of *Z. noltei* that showed an exponential recovery relationship with the irradiance of exposure (Fig. 4). When the repair was inhibited by lincomycin, no differences between control- and lincomycin-treated samples were found in *C. nodosa* in any leaf section (Fig. 4a–c). In *Z. marina*, an effect of lincomycin was found only during recovery after exposure to the highest irradiance levels in the middle and basal section (Fig. 4d–f). In contrast, in *Z. noltei*, the basal leaf section did not show any inhibitor effect (Fig. 4g), while the middle leaf section showed D1 protein repair activity only during recovery after exposure to irradiances above 45 μmol m^−2^ s^−1^ (Fig. 4h). The leaf tip of *Z. noltei* showed a significantly higher decrease in ΔF/F_m′_ after 1 h of light exposure in the presence of lincomycin, as well as a significantly lower recovery of ΔF/F_m′_ after exposure to all irradiance levels (Fig. 4i).

### Seagrass leaf anatomy and chloroplast distribution

**Figure 5:**
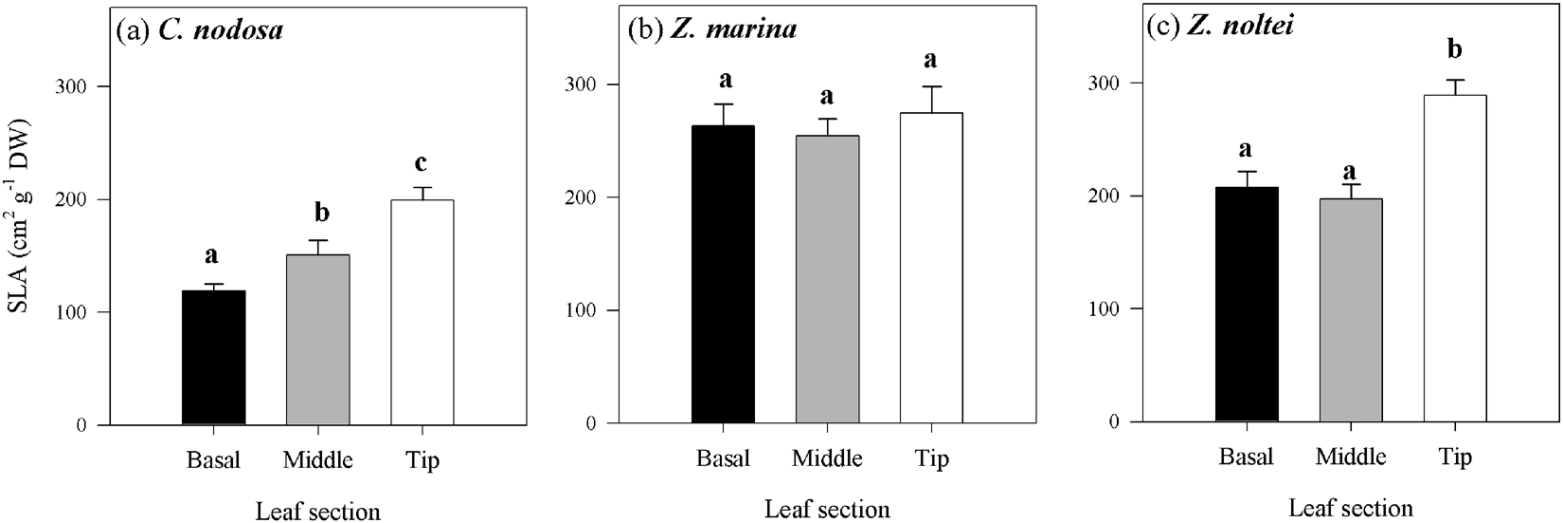
Specific leaf area (SLA) of different leaf sections of *C. nodosa* (**a**), *Z. marina* (**b**) and *Z. noltei* (**c**). Data represent mean ± SE (n = 5) and significant differences (*P* ≤ 0.05) between leaf sections are indicated by different letters.

**Figure 6:**
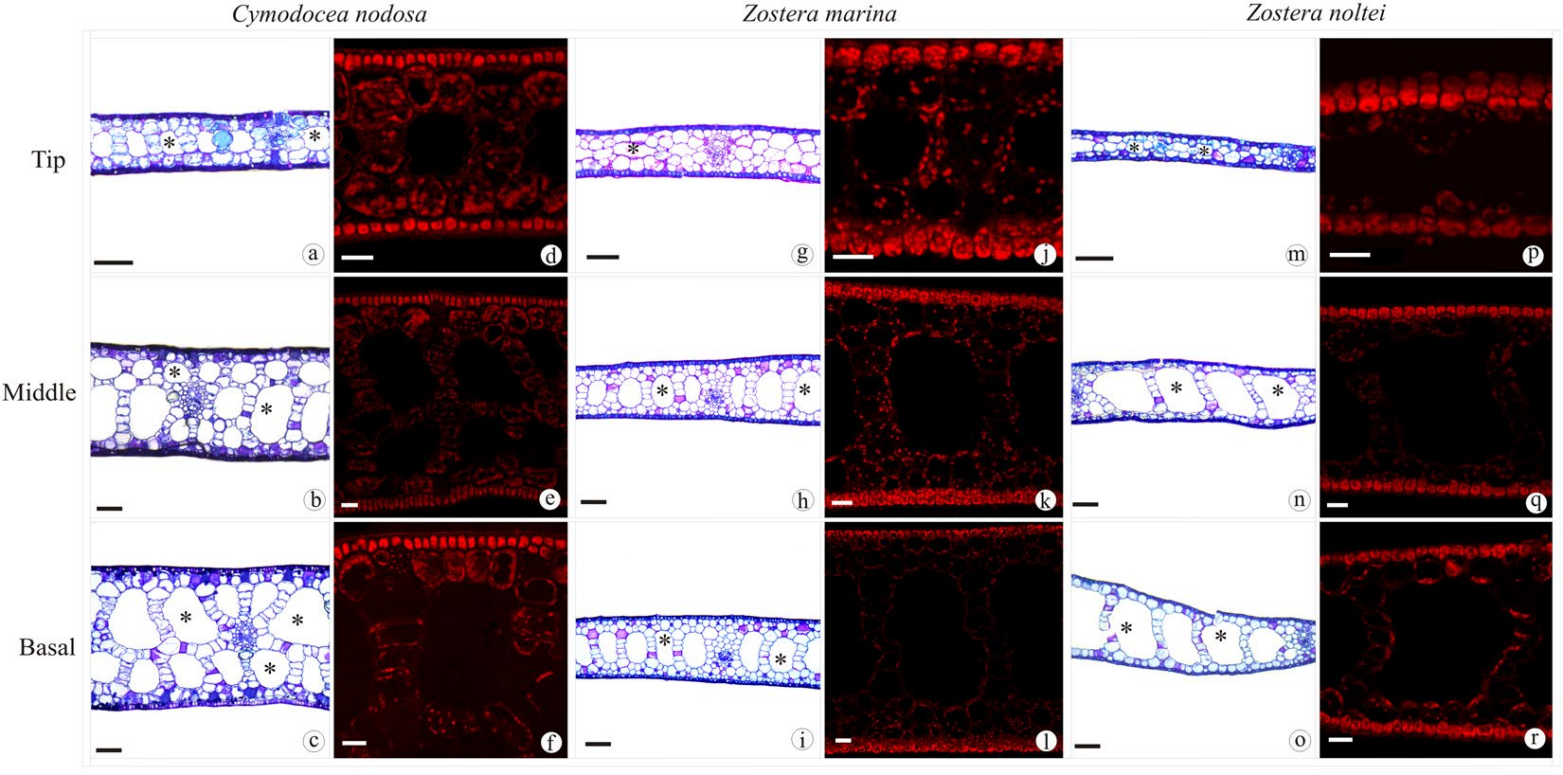
Tip, middle and basal leaf cross sections of *C. nodosa* (**a**–**f**), *Z. marina* (**g**–**l**) and *Z. noltei* (**m**–**s**). Overview on clear field stained with toluidine blue (**a**–**c**,**g**–**i**,**m**–**o**). Detail showing chloroplasts in red, emitted from 650 to 750 nm on Laser Scanning Confocal Microscope (**d**–**f**,**j**–**l**,**p**–**r**). Aerenchymas are indicated by asterisks. Scale bar: 100 µm (**a**–**c**,**g**–**i**,**m**–**o**); 25 µm (**d**–**f**,**j**–**l**,**p**–**r**).

Differences in leaf anatomy and chloroplast distribution were found among species and along individual seagrass leaves. The mean specific leaf area (SLA) was significantly lower in *C. nodosa*, compared to *Zostera* spp. (*P* < 0.0001) and differed along the leaves of the species, with exception of *Z. marina* (Fig. 5). In *C. nodosa*, SLA significantly increased from the leaf base towards the tip (Fig. 5a, *P* = 0.00024), while in *Z. noltei* SLA was similar in the basal and middle leaf section but significantly higher at the leaf tip (Fig. 5c, *P* = 0.00082). These results agree with the observations on the cross sections of the different leaf sections (see Fig. 6). The number and size of lacunae decreased towards the leaf tip in all species, with a similar pattern observed in *Zostera* spp. (Fig. 6g–h,m–o). This decrease was more drastic in *C. nodosa* due to its higher number and larger size of lacunae in the basal section, compared to the middle section and leaf tip (Fig. 6a–c). These leaf anatomical changes were accompanied by differences in the chloroplast distribution within the cells. While in all species and leaf sections the chloroplasts were concentrated in the epidermis, there were differences in the number of chloroplasts spread out in the underlying mesophyll cells. In general, a visibly higher proportion of chloroplasts seemed to be present in *C. nodosa* mesophyll cells throughout the leaf, in contrast to *Zostera* spp. Also, an increasing number of chloroplasts was concentrated in the mesophyll cells towards the leaf tip in *Z. marina* and *C. nodosa*, a trait more pronounced in the latter species (Fig. 6d–f,j–l). In contrast, in *Z. noltei* very few chloroplasts were found in the mesophyll cells, without any visible increase of their abundance toward the leaf tip (Fig. 6p–r).

### Relationship between photoacclimation and species’ life history strategies

**Figure 7:**
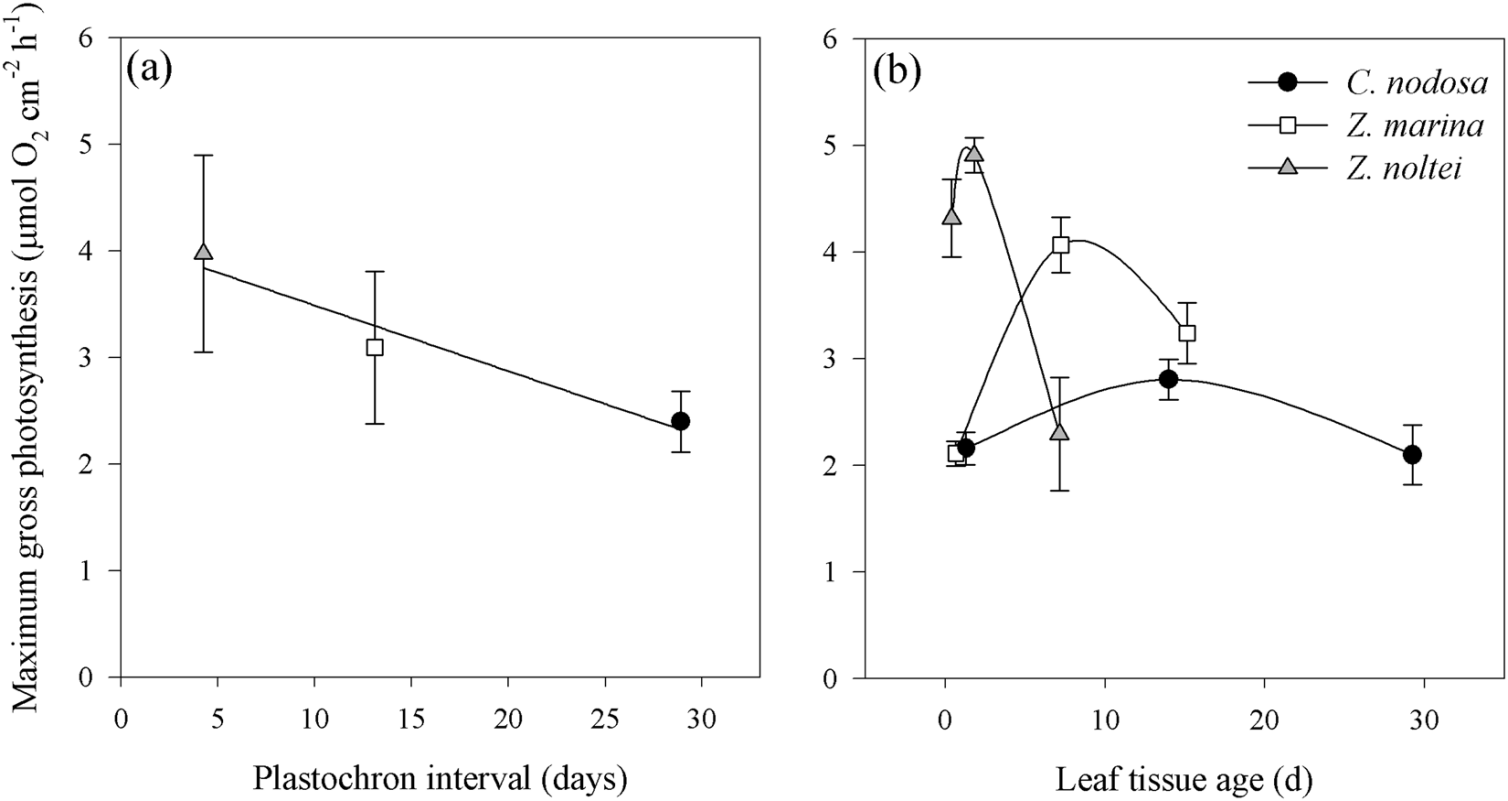
Illustration of the relationship between life history strategies and response of leaf metabolic rates to light availability in the three seagrasses studied. (**a**) Inverse linear relationship between maximum photosynthetic rate (leaf average, mean ± SE) and plastochron interval (as descriptor for leaf life span) and (**b**) comparison of chloroplast-level adjustments in relation to the specie’s leaf growth rate, here illustrated as the variation of maximum photosynthetic rates along seagrass leaves in relation to the age of the respective leaf section (see Materials and Methods).

An inverse linear relationship (R^2^ = 0.90, *P* = 0.0324) was observed between mean leaf P_max_ and leaf life span (using the plastochron interval as descriptor) (Fig. 7a).

The differences in the strength of the chloroplast-level adjustments of the species could be illustrated by relating the age of the leaf sections of each species with the variations found in their P_max_ (Fig. 7b). The fast-growing seagrass species, *Zostera* spp., invested more in these fast-response adjustments, shown by a higher variability of P_max_ along the leaf in response to light availability. In contrast, the slower-growing *C. nodosa* showed less variability along the leaf, regarding adjustments at the chloroplast level, seemingly relying more on structural adjustments at the leaf level (Fig. 6a–c).

## Discussion

Two types of photoacclimatory adjustments were observed in response to changing light levels along the leaves of the three seagrass species. While *C. nodosa* expressed rather limited physiological and biochemical adjustments in response to light availability compared to *Zostera* spp., the opposite was found with respect to leaf anatomical adjustments.

In general, the chloroplast-level acclimation to increased irradiance in seagrasses is governed by two processes: firstly, an increase in photosynthetic capacity to utilize the extra excitation energy for assimilatory processes^8,19,20^ and secondly, an increase in the capacity of photoprotective mechanisms, mainly related to xanthophyll cycling^9,21,22^. In this study, *C. nodosa* showed very limited variation in both photosynthesis (per area) and photoprotective capacity along the leaves, findings also supported by other studies^8,12,23^. On the other hand, both *Zostera* species showed differences in photosynthetic and photoprotective capacity along the leaves, even though there were lower differences between the leaf base and middle leaf section in *Z. noltei*, while in *Z. marina* significant differences among all leaf sections were found (Tables 2 and 3). This may be explained by the higher LAI of *Z. marina* and by their vertical distribution, as *Z. marina* mainly inhabits the subtidal, while *Z. noltei* is found in the intertidal zone. The upright habit of the former creates a more gradual increase in light availability along the leaf within the canopy, while the intertidal *Z. noltei* is exposed to tidal movement, causing a more horizontal position of the leaves. Hence, light availability along the leaf is less gradual, as the upper section of the leaf is exposed to high light levels, especially during low tide, while the basal leaf section is protected from stressful light conditions by the overlying canopy. This different light exposure is also reflected in the spatial variation of F_v_/F_m_ along the leaf (Fig. 1b) and might explain the fact that in *Z. noltei* the most productive leaf section was located closer to the basal section and not at the leaf middle as in the other species. No clear gradients in chloroplast-level adjustments in response to increasing light levels along the leaves were found in *Zostera* spp., as both species exhibited lower photosynthetic and photoprotective capacities at the leaf tip. In *Z. noltei*, this was related to higher photodamage at the tip, as shown by the high D1-protein turnover during and after light exposure (Fig. 4g–i), thus reducing the number of active photosystems II. This trait was only marginally present in *Z. marina* at the basal and middle leaf section, while no evidence of D1 protein repair in response to high-light stress was observed in *C. nodosa*.

There were species-specific differences in photoacclimation at the leaf level, related to changes in SLA and chloroplast distribution within epidermis and mesophyll cells. Unlike chloroplast-level adjustments, *C. nodosa* showed the largest variation in leaf-level acclimatory responses along the leaf. The leaf anatomy of this species adjusted to increasing light levels by gradually redistributing the chloroplasts in different cell layers. The SLA increase in *C. nodosa* seemed to be due to a decrease in leaf thickness, caused by reduction of number of cell layers from the leaf base towards the tip (Figs 5a and 6a–c). Similarly, in *Z. noltei* the significantly higher SLA of the leaf tip was associated with a strong decrease in leaf thickness, but without a gradual reduction in cell layers (Figs 5c and 6m–o). Such changes in SLA, directly related to a decrease in leaf thickness, agree with previous studies in *C. nodosa* and *Thalassia testudinum*^12,24^. In addition to changes in leaf anatomy, the proportion of chloroplasts in the mesophyll cells increased towards the leaf tip where the light level is higher, a feature so far only reported in *Z. capricorni*, where higher mesophyll chloroplast densities were found under high- compared to low-light acclimated leaves^10^. This study also showed that the higher chloroplast density in the mesophyll cells was accompanied by a decrease of chlorophyll per chloroplast.

The occurrence of a small proportion of chloroplasts in seagrass mesophyll cells can be found throughout the literature^25–29^, but so far, no attention has been paid to its implications. Considering the changes in leaf anatomy and the occurrence of chloroplasts in the mesophyll cells in *C. nodosa*, this trait may give the species an advantage with respect to light limitation. The lower density of chloroplasts in the mesophyll of the basal section might minimize the strong package effect caused by the concentration of chloroplasts in the epidermis^30^. This is supported by the higher optical absorption (at similar chlorophyll contents) with low spatial variation along *C. nodosa* leaves, compared to *Z. marina* (Fig. 1a), which did not show changes in SLA and had a lower proportion of its chloroplasts located in the mesophyll cells (Fig. 6). On the other hand, as light levels increase towards the leaf tip, the distribution of a higher number of chloroplasts deeper into the tissue may protect them from high-light stress, thus ensuring maintenance of leaf photosynthetic rates. In the case of *C. nodosa*, the lower SLA of the leaves compared to the other studied species, and its gradual increase towards the leaf tips, allows some regulation of the internal light field efficiently by changing the optical light path^24^. It hereby protects a fraction of the chloroplasts from excessive light levels by their wide dispersion in the epidermal layer and the mesophyll away from the leaf surface, which explains the similar high-light responses along the *C. nodosa* leaf compared to *Zostera* spp., while expressing less NPQ and xanthophyll cycling. This anatomical feature creates a heterogeneous population of chloroplasts and thus, the physiological measurements reflect an integrated response from chloroplasts at different depths within the mesophyll^31^. In contrast, the *Zostera* spp. chloroplasts occur more exclusively in the epidermal layer and little-to-no changes in SLA have been found in these species. This may explain the need for increasing photoprotective capacity and photosynthetic plasticity along the leaf in *Zostera* spp.

The assessment of distinct types of photoacclimatory adjustments along the leaves revealed clear differences in light acclimation strategies among species. *Cymodocea nodosa* seems to pursue a strategy involving a combination of leaf anatomical and, to a lesser degree, physiological and biochemical adjustments in response to different light environments. On the other hand, adjustments in *Zostera* spp. leaf anatomy are of minor importance compared to chloroplast-level responses. Differences were also found between the congeneric species, as *Z. marina* seems to adjust to increasing light levels with the distribution of a higher proportion of chloroplast into the underlying mesophyll cells (Fig. 6), thus decreasing the light the chloroplasts are exposed to, while *Z. noltei* relies mainly on physiological/biochemical adjustments. In this context, differences in the adjustments at different organizational levels have to be taken into account in interpretation of variation of leaf photoacclimatory descriptors at the chloroplast level, based on weight. In species such as *C. nodosa*, which also express leaf-anatomical changes that result in changes in biomass descriptors, it may lead to erroneous conclusions in understanding the photoacclimatory responses of seagrasses (see Table 2).

Here we revealed that the terrestrial plants well-known relationship between life history strategy and leaf metabolic rates^32,33^ also holds for seagrasses, specifically between leaf life span and P_max_ (Fig. 7a) and between the species life history traits and chloroplast-level adjustments (Fig. 7b). Considering their fast leaf elongation and turnover rates, and short leaf life spans, it is not surprising that *Z. marina* and *Z. noltei* rely more on biochemical adjustments than on leaf anatomical changes to acclimate to changing light levels. This seems to be combined with meadow/shoot-level adjustment (shoot density and height, and leaf biomass), a strategy also favored by the faster growth of these species. *Zostera marina* in particular may compensate for the lack of leaf anatomical adjustments^34^ with a higher role of acclimation responses at the canopy and shoot-level, as it has the highest reported LAI among the three species (1.7–6.7 m^2^ leaf area m^−2^ area^35^). This agrees with previous reports^36^ and is also supported by our findings of a higher LAI compared to *C. nodosa* (0.7 vs. 1.04), despite similar shoot densities. On the other hand, the importance of anatomical adjustments that act at a longer time scale in the photoacclimation of *C. nodosa* seems to be governed by its slower leaf elongation rate, lower leaf turnover and longer life span (Table 4). This kind of adjustments represents a structural investment, more justifiable than in short-lived species with a high leaf turnover, such as *Zostera* spp.

**Table 4:** Summary of reported depth ranges and leaf structural and growth parameters of the seagrass species studied (same sampling site as present study or close by: *Ria Formosa, southern Portugal and ^#^Cádiz Bay, south-western Spain; ^§^mesocosm study).

	*C. nodosa*	*Z. marina*	*Z. noltei*
Depth range	4–40m^62^ Shallow to deep subtidal (50–60 m)^63^	1–30m^62^ Mostly subtidal (10–15 m)^63^	Intertidal^63^
Shoot elongation rate (SER, cm plant^−1^ day^−1^)	0.454^64^^#§^ 0.29^65^	1.13^65^	1.18^66^^*§^ 1.13–2.84^67^^*^ 1.5–2.7^68^^*^ 0.8–2.1^69^^#^
Leaf life span (days)	45^62^–150^35^	51.4^62^–90^35^	30^35^ 6.4–11.2^67^^*^
Leaf plastochron interval (days)	28.9^70^^*^ 32.9^62^	13.1^62^	3.5–5^68^^*^ 3.4^62^
Leaf turnover (year^−1^)	3.48^62^	11.17^62^ 13.7^71^^#^	16.42^62^ 14.6–51^68^^*^
Leaf production (leaves year^−1^)	11.1^62^ 11.8–14^70^^*^	27.9^62^	107.0^62^
Leaves per shoot	2–5^63^	3–7^63^	2–5^63^
Leaf Area Index (LAI, m^2^ m^−2^)	0.2–3.5^72,73^^*#^	1.7–9^36,72^	0.2–1.9^35,73^^*#^

In summary, seagrasses with different life histories show different photoacclimation strategies, which are related to differences in the relative weight of photoacclimatory processes at the chloroplast level versus adjustments at the leaf-anatomical level. In this context, shoot- and meadow-level acclimatory responses should also be considered. This knowledge will bring us one step forward towards the understanding of how these species adjust to changing environmental conditions such as light availability, which will be critical for predicting the effects of local and global changes and accordingly develop adequate management strategies.

## Methods

### Sampling site, collection and maintenance

The Ria Formosa coastal lagoon (South Portugal) is a complex system of channels protected by a set of barrier islands, with the shallow to deep subtidal zones mainly dominated by co-occurring perennial beds of *Cymodocea nodosa* and *Zostera marina*, while *Z. noltei* forms dense beds in the muddy areas of the intertidal flats^37^.

Shoots of *C. nodosa* and *Z. marina* were collected in a shallow subtidal zone by SCUBA diving in November/December 2016 at 2 m depth (37.0029350°, −007.8224180°), while plants of *Z. noltei* were collected by hand during low tide from an intertidal meadow near the Ramalhete channel (37.0062222°, −007.9672000°). Care was taken to keep shoots intact during collection (i.e. maintaining the shoots–rhizome–root and within-ramets connectivity). Samples were transported in coolers to the laboratory at the Centre of Marine Sciences (CCMAR). The plants were maintained in 15 L aquaria equipped with independent air-pumps and situated in a plant culture/walk-in climatic chamber (FITOCLIMA 10000 THIM) until measurements were performed within 1–3 days after collection. Irradiance was adjusted to 150 μmol quanta m^−2^ s^−1^ at a constant photoperiod (12:12 h light:dark) and water temperature was maintained at 18 °C (same as in the field). This irradiance value, which was lower than the maximum irradiances to which the plants are exposed in the field, was chosen to avoid inducing any confounding additional stress^37^.

Only the 2^nd^ youngest leaves were used in this study, as they represent mature leaves that express the full acclimatory response, with minimal interference by epiphytes and/or damage/senescence accumulation^17,38^. In order to avoid intra-specific variability in the photoacclimatory leaf response due to differences in leaf length, 2^nd^ youngest leaves of similar length were chosen for measurements (30 cm long leaves for *C. nodosa* and *Z. marina*, 24 cm long leaves for *Z. noltei*). Before measurements, leaf epiphytes were carefully removed by scraping with a razor blade.

The leaf response was characterized in three segments per leaf (2.5 cm length): basal sections (0–2.5 cm from meristem), middle sections (12.5–15 cm distance from meristem in *C. nodosa* and *Z. marina*, and 4.5–7 cm distance from meristem in *Z. noltei*), which were the sections of highest F_v_/F_m_ values along the leaf, and apical sections (tip).

### Shoot density and LAI determination

To gather information about eventual *in situ* differences in self-shading within the seagrass canopy of the studied species, the shoot density was determined at each collection site by counting the number of shoots within 25 × 25 cm squares (n = 10). The total leaf area (based on one-side) per area of seagrass bed (leaf area index, LAI, m^2^ m^−2^), a descriptor of the degree of leaf packing within the canopy^39^, was determined.

### Absorptance measurements along leaves

The spatial variation in light absorption was measured every 3 cm along the leaf of the shoots (n = 20 per species), using the methodology described by Vásquez-Elizondo *et al*.^40^. Briefly, leaf absorption was measured under a LED lamp using the Diving PAM’s light sensor calibrated against a LiCor LI-190 cosine quantum sensor (LI-COR, Lincoln, NE, USA), maintaining a constant distance between light source and leaf, as well as between light sensor and leaf. The amount of light passing through the leaves was determined and later corrected for non-photosynthetic absorption, measured by using de-pigmented leaves (i.e. leaves soaked in diluted bleach for 1 h to remove photosynthetic pigments). Unfortunately, this technique could not be applied in *Z. noltei*, as its leaves were narrower than the diameter of the light sensor, and for accurate measurements, the samples must completely cover the light sensor.

### Photosynthetic performance

Photosynthesis was evaluated through Photosynthesis-Irradiance curves (P-I curves), measured with an oxygen electrode system (DW3/CB1, Hansatech, Norfolk, UK), as described in Silva *et al*.^8^. For each P-I curve, the leaf section (n = 5 per leaf section and species) of either *Z. marina* or *C. nodosa* were clipped and mounted vertically inside the measuring chamber for an even exposure to the incident light. Due to the small leaf area of *Z. noltei*, leaf sections from four independent leaves were used, mounted side by side in the incubation chamber, to ensure a good signal to noise ratio. GF/F filtered seawater (35‰), supplemented with NaHCO_3_ (final conc. 4 mM) to avoid CO_2_ limitation, was used for the incubations. During the measurements, the water in the incubation chamber was continuously stirred and the temperature was kept constant at 18 °C. For each replicate curve, ten light levels were applied sequentially, increasing from 0 to 1248 μmol quanta m^−2^ s^−1^. Each light level was imposed for approximately 10–15 min, enough time to obtain a straight line in the oxygen recording system, assumed as steady-state photosynthesis. At the beginning and the end of each light response curve, the samples were incubated in darkness, to determine the dark (R_D_) and post-illuminatory (or light) respiration rate (R_L_), respectively. Gross photosynthetic rates of the seagrass leaf sections, calculated by adding respiration rates (average of R_D_ and R_L_) to the net photosynthetic rates, were plotted against the different light intensities using an exponential fit. The maximum photosynthetic rates (P_max_) were obtained from the average maximum values above saturating irradiance. The photosynthetic quantum efficiency (α) was estimated from the initial slope of the light response curve by linear least-squares regression analysis. Irradiance of compensation (I_c_) was estimated from the ratio R_D_/α and the saturation irradiance (I_k_) was estimated as the ratio of P_max_/α. After each light response curve, the area of the leaf sections was measured and afterwards, they were dried at 60 °C for 48 h.

### Chlorophyll a fluorescence measurements

Photosystem II Chl*a* emission was measured with a pulse amplitude modulated fluorometer (Diving-PAM; Walz, Effeltrich, Germany). Nomenclature and parameter calculation was performed as in van Kooten and Snel^41^. Maximum PSII quantum efficiency was determined as F_v_/F_m_ in dark-acclimated leaves and the effective quantum yield ΔF/F_m′_ was determined during light exposure. The variable fluorescence (F_v_) is the difference between the maximum (F_m_) and the minimum (F_o_) emission. F_o_ is the fluorescence in darkness excited only by the pulse modulated measuring beam and F_m_ represents the maximum fluorescence measured in darkness when all the photochemical quenching is suppressed (first electron acceptor fully reduced) by a short saturating light pulse (0.8 s). F_m′_ represents the maximum fluorescence under ambient light.

The variation in F_v_/F_m_ along 2^nd^ youngest leaves of the three species was determined at the same day of sample collection, by taking measurements every 3 cm along the leaf (n = 20 per species). Before measurements, the leaves were cleaned from epiphytes and dark-acclimated for 2 h. Based on these measurements, the “middle section” of the 2^nd^ youngest leaf was chosen as the most active section (highest F_v_/F_m_ before decrease toward the tip) for further experiments.

Non-photochemical quenching (NPQ) of Chl*a* fluorescence was measured upon exposure to saturating light, provided by an LED lamp, in the three different leaf sections, which were previously dark-acclimated for 2 h before light exposure. The basal, middle and tip leaf sections (n = 6 per section and species) were exposed for 1 h to 360 μmol quanta m^−2^ s^−1^, corresponding to ~3I_k_ to ensure maximal NPQ induction. The sections were maintained in a temperature-controlled chamber at 18 °C and NPQ was monitored by placing the PAM optic fibre at an angle of 60° in relation to the light exposed side of the sample, maintaining this optical geometry during all measurements. A saturating pulse was applied every minute and NPQ was calculated as:

$${\rm{NPQ}}=({{\rm{F}}}_{{\rm{m}}}-{{\rm{F}}}_{{\rm{m}}^{\prime} })/{{\rm{F}}}_{{\rm{m}}^{\prime} }$$

At the end of the light exposure, the leaf sections were immediately flash-frozen in liquid nitrogen and kept at −80 °C until HPLC pigment analyses.

High-light response and recovery of ΔF/F_m′_ of the different leaf sections to 1-h exposure to five different light levels (45, 130, 260, 500, 700 μmol quanta m^−2^ s^−1^) and their subsequent recovery was measured. The light exposure was provided by LED lamps at a constant temperature of 18 °C and F_v_/F_m_ and ΔF/F_m′_ were measured after 1 h dark acclimation before light exposure and 1 h after light exposure, respectively. Subsequently, the recovery of ΔF/F_m′_ was measured after 1 h in dim-light (10–15 μmol quanta m^−2^ s^−1^).

The dependence of NPQ on xanthophyll cycling was evaluated by using dithiothreitol (DTT, Sigma Aldrich), which inhibits the de-epoxidation of violaxanthin to zeaxanthin^42^. The dependence of PSII photochemical activity on D1 protein synthesis was evaluated using lincomycin (Sigma Aldrich) that inhibits the synthesis of chloroplast-encoded proteins^43^.

The leaf sections were dark acclimated for 2 h and then incubated with either DTT (1 mM final concentration) or lincomycin (3 mM final concentration) through gentle vacuum pumping^17^ and afterwards maintained in darkness for another 30 min before the beginning of the experiment. To account for any effects of vacuum infiltration on leaf performance, control leaf sections were treated the same way, without the addition of an inhibitor.

### HPLC pigment analyses

Photosynthetic pigments were extracted as described in Abadía and Abadía^44^ with some modifications. Frozen leaf tissue was powdered in liquid nitrogen and sodium ascorbate, and extracted under low light with acetone 100% neutralized with CaCO_3_. Extracts were filtrated with 0.2 µm hydrophobic PTFE. Pigments were analyzed by isocratic high performance liquid chromatography (HPLC)^45,46^ in an Alliance Waters 2695 separation module (Milford MA, USA), with a Waters 2996 photodiode array detector and a Synergi Hydro-RP 80 Å Phenomenex 4.6 × 150 mm column (4 μm particle size) with security guard cartridges AQ C18 4 × 3.0 mm ID. Detection wavelength was set at 450 nm and pigment concentrations were calculated after calibration with commercial pigment standards (CaroteNature, Lupsingen, Switzerland, Sigma-Aldrich).

The de-epoxidation state (DPS), which represents the proportion of the xanthophyll-cycle pigment pool (XC-pool) associated with the relative increment in antheraxanthin (Ant) and zeaxanthin (Zea) relative to violaxanthin (Vio) was calculated as Zea + 0.5Ant/VAZ^47^. VAZ is the notation for the total XC-pool, which is the sum of Vio, Ant, and Zea.

### Leaf anatomy and microscopic assessment of chloroplast distribution in seagrass tissues

The leaf area displayed per unit dry mass invested (Specific Leaf Area- SLA, cm^2^ g^−1^ DW) was determined for each leaf section and species from the samples used for P-I curves as variation in SLA may indicate differences in leaf anatomy, such as the quantity of sclerenchyma. It is a common descriptor in terrestrial plant ecology, but not commonly used by seagrass ecologists, even though variations in SLA have been suggested to be of great importance to explain differences within and among species in the photoacclimatory leaf response^24,48,49^.

For the microscopic assessment of leaf anatomy, basal, middle and tip leaf segments of each species were fixed with “Kew” solution (40% seawater, 40% ethanol at 70%, 10% glycerin, 10% formalin (formol at 4%)). For determination of chloroplast distribution, histological cross sections were obtained, using a razor blade, and subsequently, the samples were photographed, using a laser scanning confocal microscope (Leica TCS SP-5, Wetzlar, Germany). Other samples, following dehydration by a series of aqueous ethanolic solutions, were infiltrated with historesin (Leica Historesin, Heidelberg, Germany) and afterwards treated with Toluidine Blue O (TB-O)^50^ for analysis of the general leaf anatomy.

### A posteriori tests

The three species differ greatly in their leaf growth rate and life span, with *C. nodosa* exhibiting the slowest growth rate and the longest life span, while *Z. noltei* leaves grow faster but have a much shorter life span (Table 4). Leaf Area Index (LAI) has been reported to be highest for *Z. marina* (1.7–6.7 m^2^ leaf area m^−2^ area), while LAI ranges for *C. nodosa* and *Z. noltei* are usually lower (0.2–3.5 and 0.2–1.9 m^2^ leaf area m^−2^ area) (Table 4).

In order to assess the relationship between photoacclimation and life history strategies of the three seagrass species, the mean leaf maximum photosynthetic capacity (P_max_) of each species was related to (1) the mean value of leaf plastochron intervals (Table 4), i.e. the number of days it takes to produce a new leaf, and (2) the age of the different leaf sections of leaf 2 (L2), considered in this study (see above). The leaf age of each section was estimated by dividing the distance of each section from the meristem by the leaf elongation rate (LER). As there are no local field data of leaf elongation rates for all three species studied, LER was calculated, using the leaf length of leaf 2 (L2) and leaf 1 (L1) and the reported plastochron interval (PI) of the species (see Table 4) as: LER = [Leaf length (L2) – Leaf length (L1)]/PI. This formula is based on leaf length (L2) being the product of LER and leaf age, where leaf age can be calculated by: PI + (leaf length of L1/growth rate).

### Statistical analyses

One-way ANOVA were used to test for differences among leaf section of each species and in the case of the parameters related to photoprotection (NPQ, XC-pool, DPS), also for differences among species. Newman Keuls Significant Difference *post hoc* tests were used to identify the statistically different groups. Homogeneity of the variance was tested a priori using Cochran’s test. All statistical analyses were run with the STATISTICA software package and considered significant with p < 0.05.
